# The Poor Man's Home.—XV

**Published:** 1897-11-27

**Authors:** 


### Modern Sociology.

### THE POOR MAN'S HOME.—XV.

### Housing in France—(*continued*).

Lyons seems to be more appreciative of the benefits of cheap flats. The "Cheap Dwellings Company" of that town has been able to pay 4 per cent. annually in dividends, besides adding to the reserve fund about 2¼ per cent. on the capital employed. This company was established in 1886 by four gentlemen, each one subscribing 50,000 francs (£2,000). Dividends were to be limited to 4 per cent. The articles of association provided that dwellings might be sold to tenants who wished to purchase; but as a rule they prefer to rent them. Apart from the difficulty some might have in gathering the purchase money, working men generally object to investing money in a house which, if they had to leave the district, might be an asset not easy to dispose of profitably. The experiment has been a financial success, there being no empty tenements and very few losses through arrears of rent. Such as have been experienced have been due chiefly to the leniency shown to tenants in cases of sickness or death. The rents charged are about 30 per cent. less than those demanded in the city by other proprietors, and yet, as we have said, the company is able, not merely to pay a reasonable dividend, but to put a substantial sum to reserve. The flats are of two or three rooms. Each kitchen has a cooking range, a coal box, and a cupboard, besides a supply of shelves There is also a clothes-press in each tenement, and the largest room is decorated with a marble mantelpiece. Occupants of three-room tenements have a private water-closet, but those who live in the smaller flats have to share them with another family. The closets are within the walls, but outside the tenements. In outward appearance, the blocks of the company have been made as like the rest of the town as possible. This is a wise precaution, for the poor are more sensitive than any other class about living in places the aspect of which might suggest that they were in any way meant as a charity. The ceilings are high throughout the whole buildings, except on the attic floor, where the height is only 8 ft. 6 in. On the ground floor it is 11½ ft., and the average is 10½ ft. The rentals of the two-room flats run from 8 to 11 francs (7s. 6d. to 9s. 2d.) a month. Three-room houses rent at from 14.50 to 21.50 francs a month (12s. 1d. to 18s.)

Lyons possesses also some very good small houses, erected by an association called "The Cottage." The name indicates the style of the buildings. The association is a branch of the Co-operative Consumers' Society. The money for the enterprise was provided, in the first place, by M. Chaurand, a private capitalist, for philanthropic reasons, but the management of the concern is carried on in accordance with business principles. Dividends are, however, limited to 5 per cent., and this amount has always been earned. The intention of the managers of "The Cottage" is to enable working men to buy their homes, although they are also willing to let them to such as do not want to become proprietors. There are two types of cottages. The first has three rooms, a cellar, a loft, and a garden extending to 1,076 ft. By paying 24 francs (about 19s. 6d.) a month, the tenant becomes in 15 years the owner of the house. A larger house, containing an extra room, but in other respects the same, costs 26 francs (£1 1s.) a month, but requires the payments to be kept up for 16 years and 5 months to make it the occupier's property. A tenant who wishes to do so may pay more than the stipulated monthly instalments, and thus obtain proprietorship sooner, but these advance payments are not received in less sums than 20 francs. In the case of a tenant dying or being compelled to leave the house before he has completed the payments for it he is regarded simply as a tenant, and a fifth of his monthly payments are given back to him, as well as any sums which he may have paid in advance. The four-fifths of his payments which the company retains is, of course, regarded as mere rental. The association thus acts as a sort of savings bank or benefit society, and the occupant is a capitalist to the extent of the money he has paid for his house. In these houses the kitchen measures 15 ft. 5 in. by 9 ft. 10 in., not counting an alcove which, if reckoned in, would add 5 ft. 3 in. to the length. The bedrooms are 13 ft. 9 in. and 10 ft. 6 in. long respectively, while their width is 9 ft. 4 in. The ceilings are 9 ft. 10 in. high.

In Rouen, also, there are some self-contained dwellings, which seem to be much more popular than the flats. The "Rouen Cheap Dwellings Company" pays, indeed, only 3 per cent. in dividends, but it is accumulating a very large reserve. If the sum thus kept back were divided among the shareholders, the total dividend would be nearer 8 per cent. As in Lyons, the tenants can become purchasers of their houses, but they can also enter them simply as tenants, without any such intention. The rent of a house of five rooms is 207 francs (£8 6s. 10d.) a year; but by paying 312 francs (£12 10s.) for sixteen years the occupant becomes its owner. The average rental for a house of a similar kind in the neighbourhood is 300 francs (£12), so that there is every inducement to a sensible man to give the other 10s., which in time makes him the landlord of his home. In these houses the kitchen is 15 ft. 8 in. long and 9 ft. 2 in. wide. There is a parlour of the same size, and a bedroom somewhat larger. The two other bedrooms measure 9 ft. 2 in. by 6 ft. 7 in. At the end of the garden attached to each house there is a building which includes a laundry, a privy (of a primitive order, the refuse simply falling into a pit which is cleaned occasionally), and a shed for storing fuel. There is no cooking range provided, but the gas company of Rouen gives gas stoves and also brackets. There is no water supply in the houses, but a fountain in the centre of the group of houses supplies all.

